# Identifying Molecular Markers of Successful Graft Union Formation and Compatibility

**DOI:** 10.3389/fpls.2020.610352

**Published:** 2020-12-02

**Authors:** Grégoire Loupit, Sarah Jane Cookson

**Affiliations:** EGFV, Univ. Bordeaux, Bordeaux Sciences Agro, INRAE, ISVV, Villenave d'Ornon, France

**Keywords:** grafting, transcripts, polyphenols, graft incompatibility, scion and rootstock, oxidative stress

## Abstract

Grafting is a technique used for millennia for vegetative propagation, especially in perennial fruit crops. This method, used on woody and herbaceous plants, can improve several agronomic characteristics, such as yield or vigor, as well as tolerance to biotic and abiotic stresses. However, some scion/rootstock combinations suffer from poor graft compatibility, i.e., they are unable to form and/or sustain a successful graft union. Identifying symptoms of graft incompatibility is difficult because they are not always present in the first years after grafting and in most cases the causes of incompatibility are still poorly understood. Studies of changes in transcript abundance during graft union formation indicate that grafting responses are similar to responses to wounding and include the differential expression of genes related to hormone signaling, oxidative stress, formation of new vascular vessels, cell development, and secondary metabolites, in particular polyphenols. This review summarizes current knowledge of the changes in transcript abundance, redox status and metabolites accumulation during graft union formation and in cases of graft incompatibility. The goal of this review is to discuss the possibility of identifying marker transcripts, enzyme activities and/or metabolites of grafting success and graft compatibility which could be used to score grafting success for genetic research and in breeding programs. We highlight gaps in current knowledge and potential research directions in this field.

## Introduction

Grafting is a traditional horticultural technique that manipulates plant wound healing mechanisms to join together two genotypes to form a composite plant. Grafting is used for different reasons: for example, to control vegetative multiplication, reduce the time to obtain the fruits, change cultivars quickly, increase or decrease the size, or provide tolerance to biotic or abiotic stresses (Mudge et al., 2009). Grafting is frequently used in the production of woody fruit crops such as citrus, figs, apples, pears, quince, and grapevine and various vegetable crops such as tomatoes, watermelons, and cucumbers. Today, thanks to the large panel of rootstocks available in many grafted plants, the scion/rootstock combination can be adapted to a type of soil, climate or production objective (for example for a certain vigor and yield).

**Figure 1:**
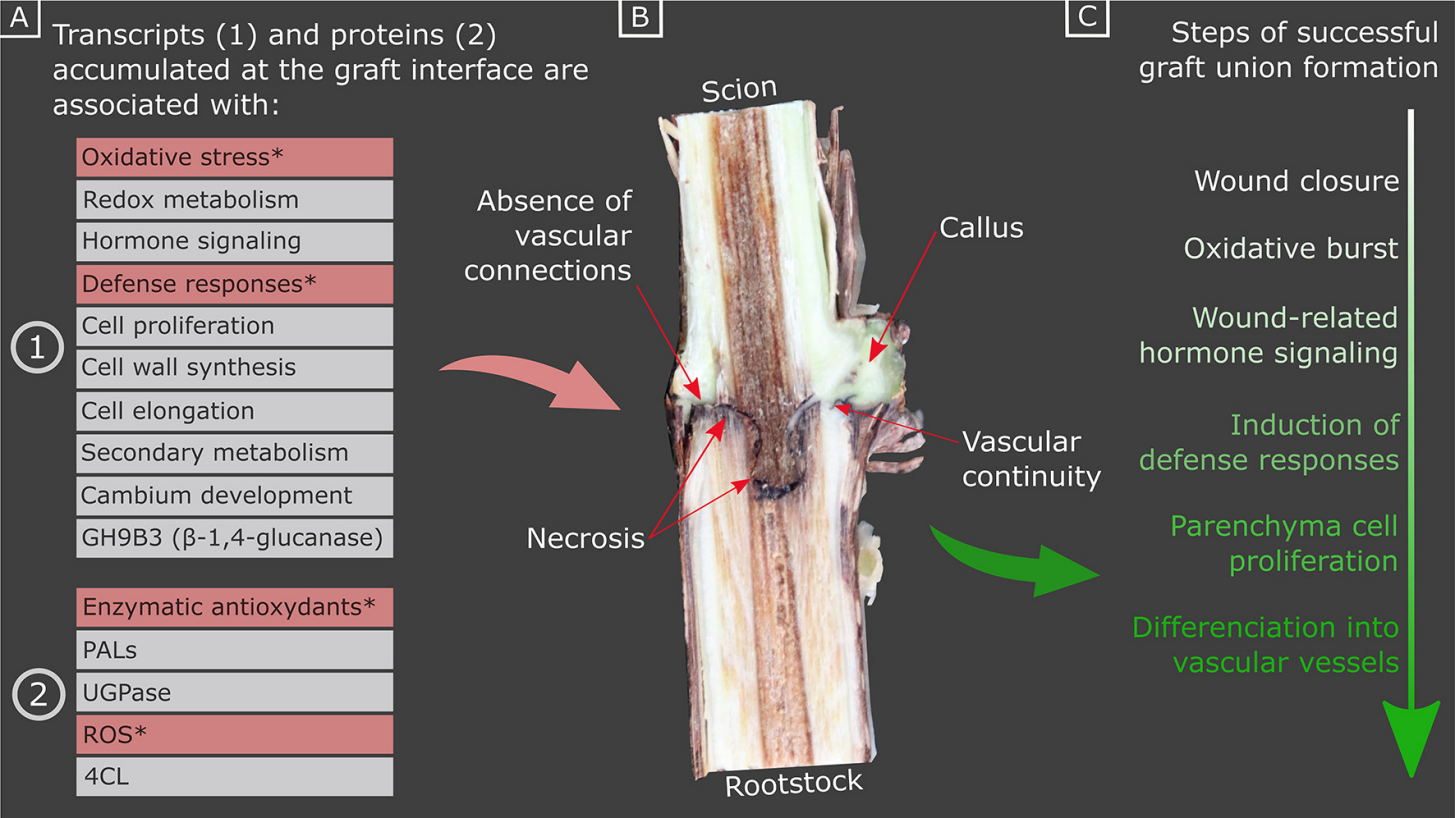
Summary of **(A)** the transcripts and proteins accumulated at the graft interface during graft union formation (stars indicate the transcripts and proteins which are more highly accumulated in hetero-grafts vs. homo-grafts, and/or incompatible vs. compatible combinations), **(B)** a photograph of a cross section of a homo-graft interface, 4 months after grafting, illustrating the appearance of necrosis, callus and vascular continuity, and **(C)** the sequence of events underlying graft union formation. PALs, PHENYLALANINE AMMONIA LYASEs; UGPase, UDP-glucose pyrophosphorylase; 4CL, 4-COUMARATE:COA LIGASE; ROS, reactive oxygen species.

We can differentiate several stages of development for the formation of a successful graft. Presumably, the first stage of graft union formation is the initial mechanical injury response (i.e., cellular damage and the disruption of the protective layers), which requires rapid wound closure to prevent water loss and pathogen entry. Polymerized phenolic compounds such as suberin and lignin accumulate to act as a physical and antimicrobial barrier at the site of wounds. Wounding triggers an oxidative stress burst, changes to metabolism, wound-related hormone signaling and the initiation of defense responses such as the induction of pathogenesis-related proteins. During graft union formation, there is a proliferation of parenchymal cells, to form the callus which will serve as a bridge between the two tissues. Then, there is the differentiation of the cambial cells into vascular vessels, which begins with the formation of phloem vessels in some herbaceous plants [from 3 days after grafting (DAG)] and then xylem vessels (Trinchera et al., 2013; Melnyk et al., 2015) and allows the connection between scion and rootstock (Figure 1C).

**Figure 2:**
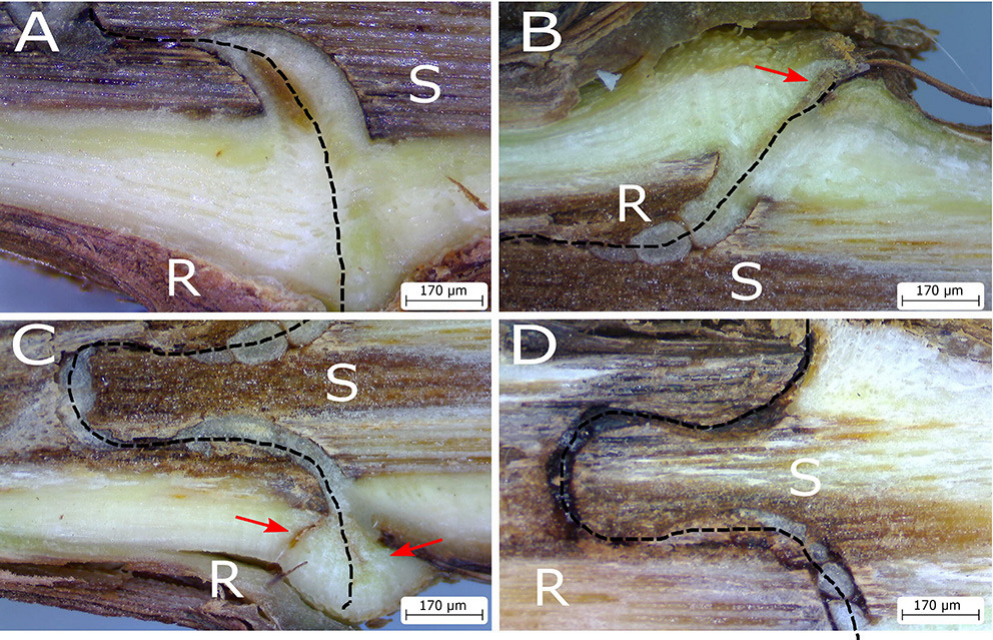
Photographs of the graft interface of homo-grafts of grapevine 4 months after grafting showing decreasing levels of tissue continuity between the scion (S) and rootstock (R). Necrosis in the callus tissue is absent in **(A)**, small amounts of necrosis are indicated in by red arrows in **(B)** and **(C)**, and poor tissue continuity in **(D)**. Graft interface indicated by a dashed line.

However, in cases of some scion/rootstock combinations, both genotypes do not always form a successful graft and the graft interface is associated with necrosis (Figure 2), which impacts the quality of the plant formed, even several years after grafting (Pina and Errea, 2005). In general, graft incompatibility increases with taxonomic distance, which most intra- and inter-specific being compatible, and most interfamilial grafts being incompatible (Goldschmidt, 2014). Graft incompatibilities have been described in many woody species such as grapevine (Sarooshi et al., 1982), pear or quince (Musacchi et al., 2000; Ciobotari et al., 2010), litchi (Chen et al., 2016), apricot (Usenik et al., 2006), and cherries (Usenik and Stampar, 2001). However, in herbaceous plants, it is possible to graft different plant families together in the short-term, such as *Brassicaceae spp*. or tomato with *Arabidopsis thaliana*, although their compatibility is limited due to poor vascular connection between the scion and rootstock (Flaishman et al., 2008). Graft-inoculation of pathogens often exploits the short-term survival of interfamilial grafts for scientific study in cases when the pathogen is not readily or not at all mechanically transmissible (Vigne et al., 2005; Aryan et al., 2016). It has recently been shown that *Nicotiana benthamiana* is very interfamily graft compatible, which is due to the ability of this species to express an extracellular β-1,4-glucanase (Notaguchi et al., 2020).

The causes of graft incompatibility are multiple; genetic proximity, poor craftsmanship, climatic conditions or pathogens can harm a successful graft union formation and maintenance as well as differences in the metabolism of the scion and the rootstock. It is difficult to know if a grafted plant will survive or die, moreover, there are few visual indicators of grafting success at an early stage of development (Figure 1B) (Tedesco et al., 2020). The identification of molecular markers of grafting success would be a great advantage for genetic research and rootstock selection programs. The objective of this review is to provide an overview of our current knowledge on the molecular mechanisms potentially involved in graft union formation and graft incompatibility with the view to identifying markers of grafting success in woody species. Although plant hormones are known to be involved in graft union formation and hormonal treatments can alter grafting success, this will not be included in this review as it has been reviewed elsewhere (Nanda and Melnyk, 2018).

## Identifying the Transcripts and Proteins Associated With Tissue Healing and Graft Union Formation in Homo-Grafts

In horticulture, hetero-grafting is used in which two different genotypes are grafted together to combine different shoot and root traits of interest. However, homo-grafting (when a genotype is grafted with a plant of the same genotype) and auto-grafting (when the same plant is grafted with itself) are only used in scientific study (e.g., Moore and Walker, 1981; Turnbull et al., 2002). In woody perennial species, changes in gene expression during homo-graft formation has been most studied in *Carya spp*. (Zheng et al., 2010; Qiu et al., 2016; Mo et al., 2018a,b). Firstly, cDNA-AFLP (complementary DNA amplified fragment length polymorphism) was used (at 0, 3, 7, and 14 DAG; Zheng et al., 2010) and subsequently RNAseq was used to quantify both mRNAs (at 0, 7, and 14 DAG; Qiu et al., 2016, and at 0, 8, 15, and 30 DAG; Mo et al., 2018b) and microRNAs (at 0, 8, 15, and 30 DAG; Mo et al., 2018a). These studies have been complemented by proteomic studies (at 7 DAG; Xu et al., 2017, and at 0, 3, 8, 15, and 30 DAG; Mo et al., 2017), which are described below. As stated above, grafting triggers wound responses such as the rapid up-regulation of the expression of genes involved in oxidative stress, wound-related hormone signaling and defense responses; these responses were also seen in the first 14 DAG in homo-grafts of *Carya spp*. (Qiu et al., 2016; Mo et al., 2018b). Another early response to grafting is the proliferation of cells at the graft interface to form a callus, as such genes involved in cell proliferation and cambium development were found to be highly expressed in the graft interface in the first 14 DAG (Zheng et al., 2010; Qiu et al., 2016; Mo et al., 2018b). The vascular connections then form between scion and rootstock to allow the long-term survival of the graft and are essential for successful graft union formation (Pina and Errea, 2005). At 30 DAG, a high expression of different genes involved in the formation of vascular tissues such as lignin metabolic processes, tubulin genes (involved in cell elongation), R2R3-type MYB transcription factors (involved in cell wall synthesis) and metacaspase genes (probably involved in the process of plant programmed cell death) was found at the graft interface of *C. illinoinensis* (Mo et al., 2018a). Unfortunately, the data from these papers has not been integrated together to give an overview of the changes occurring during graft formation in *Carya spp*. Furthermore, the results are difficult to interpret because gene expression was only quantified at the graft interface, without control scion and/or rootstock tissue samples, so it is impossible to determine which transcripts are associated with graft union formation and which are associated with plant responses to the environment or other factors. As such the results from these papers seem rather different even though they are working on the same genus using the same grafting technique, for example, in Qiu et al. (2016) 10 times more genes are differentially expressed (DE) at 7 than 14 DAG (relative to 0 DAG), whereas in Mo et al. (2018b) the number of genes DE from 0 to 8, 15, and 30 DAG increases over time. Despite these differences, genes belonging to some functional categories were DE in the graft interface tissues over time in both studies, such as categories related to metabolism, defense responses and hormone signaling (Qiu et al., 2016; Mo et al., 2018b).

Gene expression changes occurring during homo-graft formation in woody plants has also been studied in pear (although the plants were micro-grafted *in vitro*; Yang et al., 2017, and grapevine; Cookson et al., 2013). The study of Yang et al. (2017) was also only done on graft interface tissues, without the corresponding scion and/or rootstock stem controls, but as the growth conditions were controlled, it is more reasonable to assume that there was little change in gene expression in the stem over time due to factors other than grafting. However, only few genes were studied because Yang et al. (2017) used cDNA-AFLP rather than more performant transcript quantification techniques. Cookson et al. (2013) compared the transcriptomes of the rootstock wood and graft interface tissues at 3 and 28 DAG in woody homo-grafts of grapevine using whole genome microarrays. The graft union was associated with the up-regulation of gene expression, and more genes were DE at 28 than 3 DAG (Cookson et al., 2013). In agreement with the studies on *Carya spp*. described above, the genes highly expressed at the graft interface were associated with cell wall, secondary metabolism, stress, jasmonate signaling and various other signaling pathways. As grapevine grafting is done on dormant woody stem in the spring time, graft union formation coincides with the spring activation of the stem growth and metabolic activity; Cookson et al. (2013) found that far more genes were DE over time (from 3 to 28 DAG, i.e., genes associated with spring activation of growth and metabolic activity) than between the wood and graft interface (i.e., genes associated with graft union formation). Furthermore, there was a considerable overlap between those genes DE between the graft interface and the wood tissue, and those genes DE over time suggesting that similar mechanisms are involved in graft union formation and spring stem activation. This observation suggests that identifying the genes involved in graft union formation in woody perennials requires a detailed time-course, as well as scion and rootstock control samples.

There have also been a number of studies into the gene expression changes occurring during homo-graft union formation in herbaceous plants (Yin et al., 2012; Melnyk et al., 2018; Xie et al., 2019). The most complete and detailed study was done by Melnyk et al. (2018) who described the genes DE during the first 10 DAG of hypocotyl homo-grafts of *A. thaliana*; this excellent study separated the response of the scion and rootstock, and included necessary controls such as un-grafted plants, and cut, but not assembled scions and rootstocks. Melnyk et al. (2018) showed that genes associated with cambium, phloem and xylem formation are sequentially up-regulated during graft union formation and that the response of wounded tissue is different to that of grafted tissues. Furthermore, Melnyk et al. (2018) demonstrated that the response of the scion and rootstock are different; this was in part driven by the carbon gradient between the photosynthetically active scion and the carbon-starved rootstock (Melnyk et al., 2018). Genes specifically up-regulated only during grafting were probably involved in recognition mechanisms that contribute to a successful graft union formation (Melnyk et al., 2018). Studies of a similar level of detail have yet to be done in woody, perennial grafts.

The most complete proteome analysis done to date was done in homo-grafts of bottle gourd at the graft interface and in the scion just above the graft interface 7 DAG along with un-grafted controls using mass spectroscopy-based techniques (Wang et al., 2016). The graft interface was associated with the accumulation of proteins related to wound responses and defense signaling such as genes from the gene ontology (GO) groups “response to stimulus,” “phenol-containing compound metabolic process,” “oxidoreduction coenzyme metabolic process,” and “salicylic acid metabolic process.” The proteins accumulated in the scion relative to the un-grafted control were enriched in the GO terms “response to abiotic stimulus,” suggesting that grafting alters protein abundance beyond the graft interface. Proteome studies have been done on *C. cathayensis* homo-grafts using mass spectroscopy-based techniques in which the graft interface tissue 7 DAG was compared to a pool of scion and rootstock tissue harvested before grafting (Xu et al., 2017); the GO terms “defense response,” “stress response,” and “flavonoid biosynthesis” were enriched in the proteins accumulated at the graft interface. Proteomic analysis has been done using less powerful gel-based techniques on *C. illinoenis* graft interfaces at 0, 3, 8, 15, and 30 DAG, which identified the differential accumulation of proteins related to many aspects of energy metabolism and stress and defense responses; however, this study was done without scion, rootstock or un-grafted controls (Mo et al., 2017). To date, there has been no comprehensive study including scion and rootstock wood control samples of the proteome changes occurring during graft union formation in any perennial crop species.

## Identifying Transcripts and Proteins Associated With Graft Union Formation Between Different Genotypes and Associated With Graft Incompatibility

Comparison of gene expression at the graft interface during early stages of graft union formation (3, 7, 14, and 28 DAG) between two hetero-grafts and the scion homo-graft was first done in grapevine (Cookson et al., 2014). This work showed the high expression of genes involved in the stress responses at the graft interface of the hetero-grafts relative to the homo-graft control, such as, genes belonging to the functional categories pathogenesis-related proteins, polyamine oxidase, as well as several enzymes associated with oxidative stress, such as peroxidases, and enzymes involved in secondary metabolism. However, the rootstock homo-graft control sample was absent in this study (Cookson et al., 2014).

Recently, the genes DE at the graft interface between incompatible and compatible clones of the same scion variety grafted with a common rootstock was studied at 21 and 80 DAG in grapevine (Assunção et al., 2019b). At 21 DAG, genes belonging to the categories cell wall, polyamine metabolism, RNA, DNA, and signaling were more highly expressed in the compatible combination, whereas some genes related to secondary metabolism were more highly expressed in the incompatible combination (Assunção et al., 2019b). At 80 DAG, more genes were DE between the two scion/rootstock combinations than at 21 DAG. At 80 DAG, the graft interface of the least compatible combination had a higher level of expression of genes related to phenolic compounds, wound responses, hormone signaling, and galactinol synthase than the more compatible combination (Assunção et al., 2019b). It is interesting that two different clones of the same variety can behave so differently in terms of grafting success; it would be good to further characterize the differences between the clones. In particular, it is important to exclude the possibility that these differences could be due to differences in the viromes of the two clones studied. The presence of a viral agent in one of the clones could explain the lower grafting success rate, therefore, it is advisable to check for the presence of viral RNAs in RNA sequencing experiments studying hetero-grafting in the future.

The expression of genes at the graft interface of litchi has also been studied; the expression at the graft interface was compared between a compatible homo-graft and an incompatible hetero-graft at 2 h after grafting and, 14 and 21 DAG, without rootstock homo-graft, scion or rootstock wood controls (Chen et al., 2017). The expression of genes involved in the synthesis of growth-regulating hormones, such as indole-3-acetic acid, was higher at graft interface in the compatible combination compared to the incompatible combination (Chen et al., 2017). However, unlike most studies in which defense responses are more highly up-regulated in incompatible combinations, at 21 DAG, the compatible combination had a higher expression of genes involved in secondary metabolite and lignin synthesis in comparison the incompatible hetero-graft (Chen et al., 2017).

Comparative proteomic analysis of hetero- vs. homo-grafting has been done on bottle gourd 7 DAG and the hetero-graft interface was associated with the accumulation of proteins related to hydrogen peroxide (Wang et al., 2016). Other proteome studies (using gel electrophoresis-based techniques) on an *in vitro* callus graft system in *Prunus spp*. have led to the suggestion that UDP-glucose pyrophosphorylase (UGPase) could be a marker of graft compatibility, being expressed at a lower level, and in lower protein concentration and activity in incompatible callus grafts (Pina and Errea, 2008b). The high concentration of UGPase in compatible callus grafts could be indicative of a high flow of carbon to the production of metabolites, cellulose synthesis and as a consequence cellular growth.

Changes in the abundance of proteins and the expression of genes encoding enzymes of secondary metabolism are often observed in studies of graft incompatibility along with the accumulation of secondary metabolites (described below). This has led to the study of the role of PHENYLALANINE AMMONIA LYASE (PAL), the first and committed step in the phenyl propanoid pathway, in graft union formation and graft incompatibility. In *Prunus spp.*, two *PAL* genes have been identified: *ParPAL1* and *ParPAL2* (Irisarri et al., 2016). In the case of an incompatible *in vitro* callus graft, *ParPAL1* was more highly expressed at 10 and 21 DAG, and *ParPAL2* was more highly expressed at 21 DAG in comparison to compatible combinations or homo-grafts or wounded callus (Irisarri et al., 2016). This suggests that more polyphenols are produced at the graft interface of incompatible callus grafts (Irisarri et al., 2016), which is consistent with an earlier study from the same group (Pina and Errea, 2008a). However, the high expression of *PAL* at the callus graft interface in *Prunus spp*. may be restricted to these species or the experimental system. For example, although the expression of *PAL* genes was higher in some hetero-grafts of *Hevea brasiliensis*, there was no clear relationship to grafting success (Prabpree et al., 2018). Furthermore, genome-wide transcriptome studies into differences between grafting compatible vs. incompatible scion/rootstock combinations have not necessarily found the same response in other species (e.g., the expression of three *PALs* was lower at the graft interface of incompatible than compatible grafts of litchi; Chen et al., 2017). The high expression of the *4-COUMARATE:COA LIGASE/4CL*, another enzyme of phenyl propanoid synthesis, at the graft interface has also been associated with graft incompatibility in *Prunus spp*. two years after grafting (Pereira et al., 2013) and three *4CL* genes were more highly expressed at the graft interface of compatible hetero-grafts of grapevine than the homo-grafted control (from 3 to 28 DAG) (Cookson et al., 2014). However, no *4CL* genes were DE between incompatible and compatible scion/rootstock combinations of grapevine at 21 and 80 DAG (Assunção et al., 2019b).

Gene expression in the scion was recently studied in interfamilial grafts of *N. benthamiana*/Arabidopsis at 2 h after grafting, and 1, 3, 5, and 7 DAG and compared to gene expression in intact stems (Notaguchi et al., 2020). This study highlighted the increased expression of genes related to auxin signaling, cambium, xylem, phloem and provasculature development and wounding in the interfamilial hetero-graft and identified an extracellular β-1,4-glucanase involved in interfamilial grafting success. This study is important because it identified the first gene responsible for graft incompatibility, but the function of this gene is not known. It will be interesting to study the role of β-1,4-glucanases in graft union formation in the future.

Despite the differences described in the studies above, in many species common transcriptome/proteome responses have emerged as typical of a less graft compatible scion/rootstock combination: such as higher expression of genes related to stresses, wounding and secondary metabolism (Figure 1A). However, no study has been done to date including all the necessary controls to reliably identify all the genes DE during hetero-grafting and associated with graft incompatibility.

## Identifying Primary Metabolites Associated With Grafting Success and/Graft Incompatibility

Graft union formation requires cell proliferation presumably requiring the reprogramming of the primary metabolism. Grafting with photosynthetically active tissues (such as hypocotyl grafting in herbaceous or *in vitro* micro-grafting in perennial plants) rapidly results in the formation of a strong carbon gradient from the carbon-rich scion to the carbon-starved rootstock, which disappears once the phloem has reconnected. This asymmetry triggers the differential expression of the sugar-responsive genes above and below the graft union (Melnyk et al., 2018; Xie et al., 2019). Presumably, this asymmetry of carbon immediately after grafting is absent in non-photosynthetic, dormant woody perennial grafts rich in carbon reserves. Recently, the metabolite profile of the graft interface, and scion and rootstock wood of woody grafts of grapevine was studied 28 DAG: the starch content at the graft interface was lower than the surrounding woody tissues, whereas the concentration of glucose was higher (Prodhomme et al., 2019). The concentration of histidine, threonine, arginine, tyrosine, lysine, and phenylalanine was lower, and the concentration of glutamine, γ-aminobutyric acid and total proteins was higher at the graft interface compared to the surrounding tissues (Prodhomme et al., 2019). These differences in the concentration of primary metabolites between the graft interface and surrounding woody tissues are presumably related to the formation of callus cells at the graft interface, which require the mobilization of store reserves (such as starch and arginine) and are typically rich in proteins, glutamine and γ-aminobutyric acid (Prodhomme et al., 2019). To date there have been no studies linking primary metabolite profile of the graft interface and grafting success in any species.

**Figure 3:**
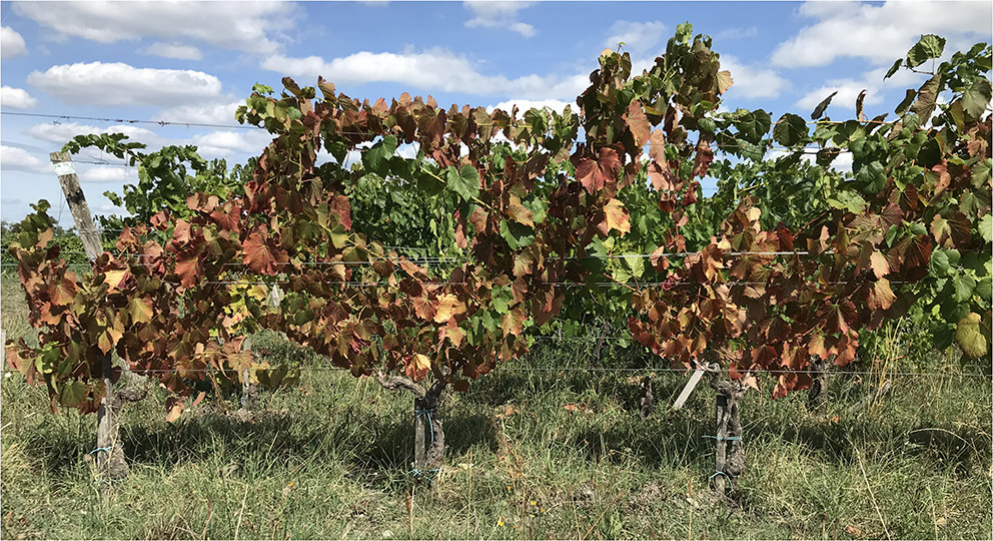
Photograph of grafted grapevines showing symptoms of graft incompatibility 10 years after grafting.

In addition to short-term modification of primary metabolite profile during graft union formation, many months to years after grafting, the accumulation of carbon in the scion is often associated with graft incompatibility and poor phloem functioning (Moing et al., 1990; Moing and Gaudillère, 1992; Ermel et al., 1999). Although not necessarily documented in the scientific literature, graft incompatibility in the field is often associated with reddening of leaves earlier at the end of the growing season than the other compatible combinations, which is also indicative of accumulation of carbon in the scion (Figure 3). However, these observations were not found in a study of four varieties of pear grafted onto two rootstocks (compatible and incompatible) (Ciobotari et al., 2010) and in citrus chip-budded trees (Mendel and Cohen, 1967).

## Identifying Redox Markers of Grafting Success and/or Graft Incompatibility

Grafting induces a response to wounding and therefore results in the generation of reactive oxygen species (ROS), such as singlet oxygen, superoxide, hydrogen peroxide, and hydroxyl radicals, which can cause severe damage to cell structure and functions. Plant have complex antioxidant system to control ROS via non-enzymatic (such as carotenoids, tocopherols, flavonoids, ascorbate, glutathione and proline) and enzymatic antioxidants [such as superoxide dismutase (SOD), catalase (CAT), glutathione reductase (GR), dehydroascorbate reductase (DHR), and ascorbate peroxidase (APX)]. The high expression of genes (Cookson et al., 2013; Assunção et al., 2019b; Xie et al., 2019) and the accumulation of proteins (Wang et al., 2016; Xu et al., 2017) associated with different elements of the antioxidant system is frequently observed at the graft interface in homo-grafts. Some studies have measured the activity of certain antioxidant enzymes during homo-graft formation, but frequently not at the graft interface itself or without adequate intact plants or stem control samples (Fernandez-Garcia et al., 2004; Miao et al., 2019).

The idea that hetero-grafting together different genotypes results in an increased level of oxidative stress at the graft interface has been supported by gene expression (Cookson et al., 2014; Assunção et al., 2019b) and proteomic studies (Wang et al., 2016; Xu et al., 2017). Some studies have measured the activity of antioxidant enzymes at the graft interface of different scion/rootstock combinations. For example, APX, DHR, GR, SOD, and CAT activities are higher in microcalli floating on suspensions quince (which is normally graft incompatible with pear) relative to the pear control suspensions (Nocito et al., 2010). Peroxidase activity at the graft interface of incompatible grafts is often higher than that of compatible grafts e.g., in *Prunus spp*. at 4 and 8 months after grafting (Zarrouk et al., 2010), in micro-grafts of eucalyptus (De Cooman et al., 1996) and in pepper/tomato grafts (Deloire and Hébant, 1982). Although SOD activity was higher at the graft interface 24 DAG in incompatible hetero-grafts of melon/Cucurbita rootstocks in comparison to compatible hetero-grafts, cell wall and soluble peroxidase activity was lower in the incompatible combination (Aloni et al., 2008). However, homo-graft rootstocks were missing from the study of Aloni et al. (2008).

To date there have been few studies quantifying ROS themselves at the graft interface during graft union formation except the study of Aloni et al. (2008), which found high hydrogen peroxide concentration at the graft interface of an incompatible scion/rootstock combination in comparison to intact scions, homo-grafted scions and hetero-grafted compatible plants at 24 DAG. The study of Aloni et al. (2008) also showed that hydrogen peroxide and the activities of antioxidant enzymes changed over time suggesting that a time course should be studied. Some studies have also examined the presence of ROS at the graft interface using histological analysis (Aloni et al., 2008; Irisarri et al., 2015). We suggest that quantifying the oxidative status of the graft interface over time in different homo- and hetero-grafts should be a priority for future research.

## Identifying Secondary Metabolites Associated With Grafting Success and/Graft Incompatibility

Many studies have highlighted the accumulation of phenolic compounds in the graft interface (Table 1), which play a role in defense responses as well as processes such as cell division, development and differentiation (Gainza et al., 2015; Pina et al., 2017).

**Table 1:** Summary of secondary metabolites accumulated at the graft interface of incompatible scion/rootstock combinations in the literature.

**Compounds**	**Species studied**	***In vitro* experimentation**	**Time after grafting**	**References**
4-Hydroxyphenylacetic acid	Olive	No	1 years	Azimi et al., 2016
Arbutin	Pear	No	4 years	Hudina et al., 2014
Catechin	Sugar plum	No	3 years	Mng'omba et al., 2008
Catechin	Apricot	No	1 years	Usenik et al., 2006
Catechin	Pear/quince	No	2 years	Musacchi et al., 2000
Catechin	Pear	No	4 years	Hudina et al., 2014
Catechin	Grapevine	No	1 month	Canas et al., 2015
Catechin	Grapevine	No	End of rooting stage	Assunção et al., 2019a
Ellagic acid	Eucalyptus	Yes	5 years	De Cooman et al., 1996
Epicatechin	Pear/quince	No	2 years	Musacchi et al., 2000
Epicatechin	Grapevine	No	3 month	Assunção et al., 2016
Ferulic acid	Olive	No	1 year	Azimi et al., 2016
Ferulic acid	Grapevine	No	3 months	Assunção et al., 2016
Flavonoids dimers	Pear/quince	No	2 years	Musacchi et al., 2000
Flavonoids dimers	Pear	No	4 years	Hudina et al., 2014
Gallic acid	Grapevine	No	1 month	Canas et al., 2015
Gallic acid	Grapevine	No	End of rooting stage	Assunção et al., 2019a
Gallic acid	Eucalyptus	Yes	5 years	De Cooman et al., 1996
Gentisic acid	Eucalyptus	Yes	5 years	De Cooman et al., 1996
p-coumaric acid	Sugar plum	No	3 years	Mng'omba et al., 2008
p-coumaric acid	Apricot	No	1 year	Usenik et al., 2006
Prunasin	Pear/quince	No	5 years	Gur et al., 1968
Sinapic acid	Grapevine	No	1 month	Canas et al., 2015
Sinapic acid	Grapevine	No	End of rooting stage	Assunção et al., 2019a

The first step in the synthesis of phenolic compounds is the conversion of the amino acid phenylalanine to ammonia and trans-cinnamic acid by PAL. Although the expression of *PAL* genes has been quantified in a number of studies of graft union formation and graft incompatibility (cited above), only one study has quantified PAL activity at the graft interface, which was 2-fold higher than to the surrounding woody tissues 28 DAG in homo-grafts of grapevine (Prodhomme et al., 2019). This high PAL activity was associated with the halving of the concentration of phenylalanine and high polyphenol concentration (Prodhomme et al., 2019). The high concentration of polyphenols at the graft interface was largely due to the accumulation of stilbenes; as the concentration of many flavanols (particularly epicatechin) was lower at the graft interface relative to the surrounding woody tissues. The low epicatechin concentration at the graft interface is in agreement with another study of graft union formation in grapevine (Canas et al., 2015). An accumulation of stilbenes in response to grafting is in agreement with a study of mechanical wounding in leaves (Chitarrini et al., 2017). Stilbenes are a particular family of molecules found only in some species of the plant kingdom; most of the studies about these compounds have focused on few species such as the grapevine or pine trees (Parage, 2013). These compounds play a protective role in plants and are involved in wound responses thanks to their antioxidant and antifungal properties (Chong et al., 2009). They also serve as signals for growth regulation (jasmonate biosynthesis), nutrition or photosynthesis. For example, certain polymers can attach to the cell wall to reinforce it following an infection (Chong et al., 2009; Marques et al., 2009). Thanks to numerous studies characterizing high concentrations of stilbenes in grapevine canes (Pawlus et al., 2013; Guerrero et al., 2016; Billet et al., 2018; Loupit et al., 2020), it is advisable to include the measurement of stilbenes in studies of graft union formation in grapevine.

The concentration of some phenolic compounds was compared at the graft interface of dormant grafts (1 year after grafting) of clones of *Vitis vinifera* cv. Syrah that are reported to be either susceptible or non-susceptible to dieback in the field many years after grafting (Canas et al., 2015). The concentration of the phenolic compounds measured differed between the scion, rootstock and graft interface tissues, and some metabolites were potentially associated with the dieback phenotype: e.g., sinapic acid was at a lower and gallic acid was at a higher concentration at the graft interface of the grafts with the Syrah clone susceptible to dieback. The same group confirmed these results in another study, including the measurement of some additional metabolites and stages of graft union development (Assunção et al., 2016). However, in both these studies data relative to actual grafting success and dieback phenotypes were absent. In another study, the metabolite profiles of graft interface, and scion and rootstock wood of two clones of grapevine grafted with a common rootstock were compared at three time points (Assunção et al., 2019a). The clone with the lower level of grafting success 3 years after grafting was associated with higher concentrations of sinapic acid at the end of the growth cycle, high concentrations of catechin during the rooting phase (after 28 DAG), and a lower concentrations of caffeic acid than the more compatible clone (Assunção et al., 2019a). The metabolites associated with grafting success (Assunção et al., 2019a) and long-term dieback (Canas et al., 2015; Assunção et al., 2016) in these studies appear to be quite different suggesting that different metabolites are involved in these two types of incompatibility responses. In addition, the work of Assunção et al. (2019a) shows that the metabolite profile of the scion, rootstock and graft interface differs at different times after grafting suggesting that metabolites markers of graft incompatibility will be specific to different stages of graft union formation.

The most famous example of a secondary metabolite involved in graft incompatibility is prunasin, which has been long known to be responsible for graft incompatibility in pear/quince grafts (Gur et al., 1968). Prunasin, a cyanogenic glycoside present in quince can move a short distance to the pear scion where it is hydrolyzed by a glucosidase resulting in the release of toxic hydrocyanic acid/cyanide, which damages cells and vessels at the graft interface and induces graft incompatibility (Gur et al., 1968). As prunasin is relative immobile, graft incompatibility in pear/quince grafts can be overcome by grafting with a compatible interstock between the pear and quince (Hartmann et al., 2011). Pear genotypes differ in their sensitivity to grafting with quince rootstocks and the concentration of other polyphenols has been studied in different pear/quince combinations. Epicatechin and procyanidin B1 were at higher, and catechin was at lower concentrations in the stem/bark at the graft interface in comparison to the surrounding woody tissues of some pear/quince and pear/pear combinations 2 years after grafting (Musacchi et al., 2000). The accumulation of procyanidin B2 at the graft interface seemed to be higher in the compatible pear/pear grafts, but no clear incompatibly marker could be identified (Musacchi et al., 2000). Another study on pear/quince incompatibility measured polyphenol concentration in the bark/phloem above and below the graft interface of three scions grafted onto five different rootstocks (and self-rooted controls) 4 years after grafting (Hudina et al., 2014). Hudina et al. (2014) showed in the rootstock of the most incompatible combination had the highest concentration of catechin, procyanidin B1 and B2, and arbutin (a compound found in *Vaccinium spp*. and pear trees), but this did not affect scion metabolite concentrations (Hudina et al., 2014).

Several phenolic compounds, such as gallic acid, gentisic acid, ellagic acid, p-coumaric acid, catechin, and quercetin-3-glucoside, were found in high concentration in less compatible combinations compared to more compatible combinations in micro-grafts of *Eucalyptus gunnii* 20 DAG (De Cooman et al., 1996). However, scion and rootstock tissues, and homo-grafted controls were absent from this study making these results difficult to interpret.

In both cherries and apricots rootstock polyphenol concentration differs between different genotypes and it seems like the rootstock alters scion polyphenol concentrations just above the graft union (Usenik and Stampar, 2001; Usenik et al., 2006), however, rootstock induced differences in scion metabolite profile was not tested statistically in these papers.

Total soluble and cell wall bound phenol concentrations were studied at the graft interface of homo- and hetero-grafts of *Uapaca kirkiana* 3 years after grafting; both total soluble and cell wall bound phenol concentrations were higher at the graft interface than the surrounding woody tissues for some, but not all, of the homo- and hetero-grafts studied (Mng'omba et al., 2008). Total soluble and cell wall bound phenol concentrations also appeared to differ between the different genotypes studied (Mng'omba et al., 2008).

In addition to the quantification of metabolites in bulk samples, the accumulation of polyphenols at the graft interface has been studied using imaging methods; the visualization of necrosis areas and callus development provides knowledge of the tissue specific location of different metabolites. For example, flavonoid accumulation has been studied in callus grafts (Pina and Errea, 2008a) and phenols in *U. kirkiana* grafts (Mng'omba et al., 2008). The exact localization of phenolic compounds at the graft interface remains to be elucidated. The use of state-of-the-art analysis methods could provide new insights into tissue level metabolites profiles at the graft interface, such as metabolite imaging using Matrix-Assisted Laser Desorption/Ionization Mass Spectrometry imaging (MALDI-MS imaging), which has previously been used in plant samples to visualize proteins (Grassl et al., 2011), primary metabolites such as sugars (Horikawa et al., 2019) and stilbenes (Becker et al., 2014).

As described above, there have been a number of studies into the accumulation of phenolic compounds associated with graft union formation and these studies have highlighted the complexity of the metabolite responses of different species. It is difficult to make generalities about the metabolite response to grafting because different samples have been taken, such as, entire stems vs. just the phloem, cambium and/or bark. Many of the results in the literature are difficult to interpret because control samples are missing. Similarly the metabolite profile of woody tissues appears to change over time suggesting that a marker of graft incompatibility may only be valid at a certain developmental stage.

## Discussion

It is known that graft incompatibility is under genetic control (Salesses and Al Kaï, 1985; Salesses and Bonnet, 1992) and the parentage of a given genotype is frequently a likely indicator of its compatibility (Cordeau, 1998). In general, to identify the genetic basis of traits of interest a range of different genetic approaches such as genome-wide association and quantitative trait loci mapping can be used. However, phenotyping graft compatibility in large populations is challenging as it requires grafting many (hundreds) individuals to accurately score this trait, which has many logistical problems. To begin to overcome these problems, recently graft union formation was assessed at 1 month and 1 year after grafting by scoring the necrotic line, and wood and bark discontinuity, and cellular arrangements at the interface in a bi-parental F1 apricot scion population grafted onto a plum rootstock (Irisarri et al., 2019). Continuous variation was found in the graft union traits scored (Irisarri et al., 2019); this study paves the way for further studies into the genetic control of graft union formation. However, understanding the genetic basis of graft incompatibility would be greatly accelerated if marker metabolites or transcripts could be identified. As outlined above, many studies have tentatively identified transcript/metabolite or enzyme activity markers of grafting success and graft incompatibility, but frequently these studies lack the control samples required to unequivocally identify marker metabolites; this should be a priority for future research. Identifying robust transcript or metabolite markers of successful graft union formation and/or graft incompatibility is challenging largely because these studies require so many control samples: the ideal experimental design would include compatible and incompatible hetero-grafts, homo-graft controls for all genotypes used, wounded controls for all genotypes used, rootstock and scion wood, graft interface samples, and intact plants. Also, it would be interesting to specify the analysis of different tissues to differentiate the grafting and/or incompatibility responses of the scion, rootstock, interface and callus tissues. Furthermore, in woody plants, grafting generally coincides with the end of dormancy in spring, which makes interpretations more complicated and requires a time-course experiment, once marker transcripts or metabolites have been identified this may suggest that their use will be restricted to certain time points after grafting.

## Author Contributions

GL drafted and SC revised the manuscript. All authors contributed to the article and approved the submitted version.

## Conflict of Interest

The authors declare that the research was conducted in the absence of any commercial or financial relationships that could be construed as a potential conflict of interest.
